# Secular trends in serum lipid levels of a Middle Eastern adult population; 10 years follow up in Tehran lipid and glucose study

**DOI:** 10.1186/1476-511X-13-20

**Published:** 2014-01-23

**Authors:** Masoumeh Kheirandish, Samaneh Asgari, Mojtaba Lotfaliany, Mohammadreza Bozorgmanesh, Navid Saadat, Maryam Tohidi, Fereidoun Azizi, Farzad Hadaegh

**Affiliations:** 1 Prevention of Metabolic Disorders Research Center, Research Institute for Endocrine Science, Shahid Beheshti University of Medical Sciences, #24, Parvaneh st, Yemen st, Chamran Exp, P.O. Box: 19395–4763, Tehran, Islamic Republic of Iran; 2 Endocrine Research Center, Research Institute for Endocrine sciences, Shahid Beheshti University of Medical Sciences, Tehran, Iran

**Keywords:** Secular trends, Serum lipids measures, Middle Eastern adult population, Cohort, Dislipidemia

## Abstract

**Background:**

To examine trends in the population levels of serum lipids among a Middle-Eastern adult population with high prevalence of dyslipidemia.

**Methods:**

A population-based cohort of adult Iranian participants, aged ≥20 years underwent four consecutive examinations between 1999–2001 and 2008–2011. Trends in age and multivariate-adjusted mean lipid levels were calculated using generalized estimating equations.

**Results:**

At each of the 4 assessments, there were significant decreases in levels of total cholesterol (TC) (multivariate-adjusted means, 5.21 vs. 4.88 mmol/L in men; 5.42 vs. 5.07 mmol/L in women), triglycerides (TGs) (2.11 vs. 1.94 mmol/L in men; 1.88 vs. 1.74 mmol/L in women), and an increase in HDL-C level in both genders (0.95 vs. 1.058 mmol/L in men; 1.103 vs. 1.246 mmol/L in women) in multivariate analyses (all Ps <0.001); however, body mass index (BMI) significantly increased simultaneously (25.92 vs. 27.45 kg/m2 in men; 27.76 vs. 30.02 kg/m2 in women) (P < 0.001). There were significant (P < 0.001) increases in fasting plasma glucose (FPG) levels only among men (5.35 vs. 5.73 mmol/L). Results did not change after excluding participants that had cardiovascular disease or used lipid lowering drugs during follow-up. There were significant decreases in the prevalence of hypercholesterolemia, low HDL-C, hypertriglyceridemia (all Ps <0.001) during follow-up. Furthermore, the consumption of lipid lowering drugs significantly increased (P <0.001).

**Conclusion:**

During a 10 years follow-up, favorable trends were observed in the population levels of TC, triglycerides, HDL-C, which could not be fully accounted for by the increase observed in the consumption of lipid lowering drugs. These favorable trends were counterbalanced by the progressive increase in general obesity and FPG level.

## Background

Coronary heart disease (CHD) is one of the main causes of mortality and morbidity worldwide, leading severe concern that CHD will become pandemic problem [1]. Cardiovascular disease (CVD) account for 38–50% of deaths in Iran [2]. Numerous studies have reported that high serum cholesterol and low high density lipoprotein cholesterol (HDL-C) are major risk factors for coronary heart disease [3,4]. There are also evidences to show that high triglycerides and CHD have an association [5,6]. The prevalence of dyslipidemia between Tehranian adult population has been reported to be high [7]. We have recently observed that all lipid measures were significant predictors of incident CHD among an Iranian population aged ≥50 years in sex and multivariate-adjusted regression models [8].

Trends in a lipid measure vary across different countries; even in the same country different lipid measures might have different trends in both directions and magnitudes of changes. A number of studies have examined the trends in serum lipid levels either in repeated cross-sectional time-series [9-15] or in longitudinal cohort studies [16-18]. It seems that the trends in the levels of total and low density lipoprotein cholesterol (LDL-C) are favorable in most of countries, except India, China and Japan [9,10,12]. In a global study of trends in serum total cholesterol in 199 countries, the mean level of total cholesterol changed little between 1980 and 2008, declining by less than 0.1 mmol/L per decade in men and women. Total cholesterol decreased in the high-income region consisting of Australasia, North America, and western Europe, and in central and eastern Europe while it increased in east and southeast Asia and Pacific [19]. In a previous report on time-trends in lipid measures among an Iranian adult population, we observed short-term favorable trends paralleling the increasing trend in obesity measures, whether such favorable trends have extended to a longer time-frame in the light of increases observed in the diabetes and obesity remains to be elucidated [15,20].

Recently the fourth follow-up assessment of a large community-based longitudinal study of a Middle East population, the Tehran Lipid and Glucose Study (TLGS) has been completed, herein, the trends in the population levels of serum lipids and indices as well as trends in prevalence of dyslipidemia among TLGS’ participants followed for more than a decade, enabling us to investigate the trends in lipid measures during the last decade.

## Methods

### Study design and sample

Detailed descriptions of TLGS have been reported elsewhere [21]. In brief, the TLGS is a large scale, long term, community-based prospective study performed on a representative sample of residents of district 13 of Tehran, the capital of Iran. The TLGS has two major components: a cross-sectional prevalence study of non-communicable disease and associated risk factors, implemented between March 1999 and December 2001, and a prospective follow-up study. Data collection is ongoing, designed to continue for at least 20 years, on triennial basis.

### Study population

**Figure 1:**
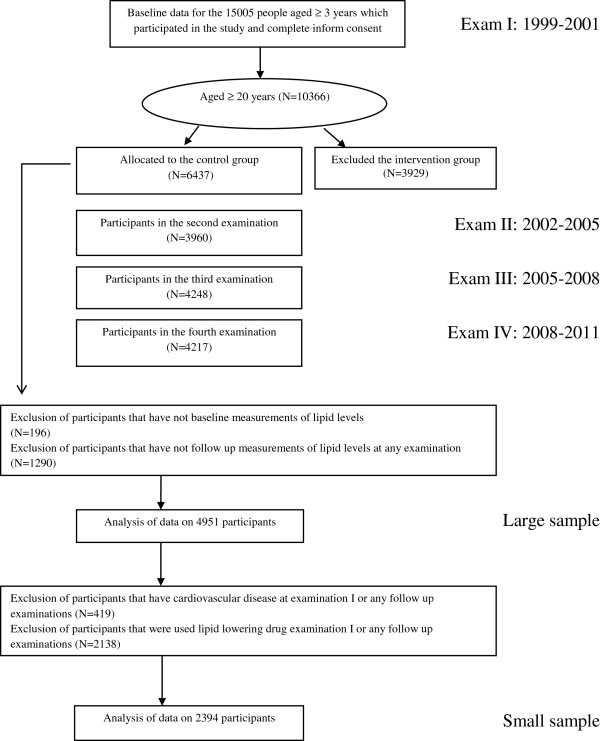
Outline of selection design of study participants.

Of total of 27340 residents aged ≥ 3 years invited by telephone call, 15005 residents participated in first examination. Of this population those aged ≥ 20 years (n=10366), were categorized into the cohort (n=6437) and intervention group (n=3929), the latter to be educated for implementation of life style modifications. After excluding intervention group, participants without any lipid levels record in baseline (n=196), and participants without follow-up record in any examination (n=1290), the final sample consisted of a total of 4951 individuals (2866 women) with at least 1 follow-up. In the secondary analyses, we analyzed trends of lipid levels in a smaller sample (2394) obtained by excluding individuals with prevalent or incident cardiovascular disease (CVD) and current use of lipid lowering drugs (Figure 1).

The design of the study was approved by the Ethics Committee of the Research Institute for Endocrine Sciences, Shahid Beheshti University of Medical Sciences, and all participants provided written informed consent.

### Clinical and anthropometric measurements

Subjects were interviewed by trained interviewers using pretested questionnaires. Information on age, sex, past medical history of CVD, medication use, smoking habits and family history of premature CVD was collected. Anthropometric measured including weight and waist circumference (WC). Using standard protocols, WC was measured by a trained individual at the level of the umbilicus. Body mass index (BMI) was calculated as weight in kilograms divided by height in squared meters. Blood pressure (BP) was measured twice in a seated position after 15 min resting using a standard mercury sphygmomanometer. Education status was categorized into 3 groups: 1. Illiterate/primary school (less than 6 years); 2. Below diploma /diploma (6-12 years) and 3. Higher than diploma (more than 12 years). Marital status was categorized as single, married and widowed/divorced.

### Laboratory measurements

After 12–14 h overnight fasting, blood samples were drawn from veins of the participitants into Vacutainer® tubes between 7.00 and 9.00 A.M. and centrifuged within 30–45 min of collection. Fasting plasma glucose (FPG) was measured by the enzymatic colorimetric glucose oxidase method; inter-and intra-assay coefficients of variation (CV) at baseline and follow-up phases were both less than 2.3%. A Selectra 2 autoanalyzer (Vital Scientific, Spankeren, Netherlands) was used in the TLGS research laboratory, on the day of blood collection, to analyze samples for serum total cholesterol (TC) and TGs. Enzymatic colorimetric tests were used to assay TC with cholesterol esterase and cholesterol oxidase; for TGs, glycerol phosphate oxidase was used. HDL-C was measured after precipitation of the lipoprotein-B-containing lipoproteins with phosphotungstic acid. LDL-C was calculated according to the Friedewald formula if TGs were <4.5 mmol/l [22]. Non-HDL-C was calculated by subtracting HDL-C from TC; TC/HDL-C and TG/HDL-C were calculated by dividing TC and TG by HDL-C, respectively. Both inter and intra-assay coefficients of variation were less than 1.9, 2.1 and 3% for TC, TGs and HDL-C, respectively in all baseline and follow-up assays of lipid profile.

### Cardiovascular disease outcome

Details of the collection of cardiovascular outcome data have been published elsewhere [21]. In the current study, the events targeted were the first CVD events, including definite myocardial infarction (MI), probable MI, unstable angina, angiographic-proven coronary heart disease (CHD), and stroke (as defined by a new neurological deficit that lasted more than 24 h).

### Definition of terms

Dyslipidemia was defined as follows: Hypercholesterolemia: serum TC ≥ 6.19 mmol/L; Hypertriglyceridemia: serum TGs ≥ 2.26 mmol/L; Low HDL-C: serum HDL < 1.036 mmol/L; High non-HDL-C: serum non-HDL-C ≥ 5.15 mmol/L; High TC/HDL-C ≥ 5.97; High TG /HDL-C ≥ 2.18 [23].

Participants who had systolic blood pressure ≥ 140 mmHg or diastolic blood pressure ≥ 90 mmHg or were on antihypertensive drugs were referred to as hypertensive. Diabetes mellitus was ascertained among participants who had FPG ≥7 mmol/L or were on glucose lowering medication. Participants who were smoking at the time of examination were considered as current smokers.

### Statistical analysis

Continuously-distributed variables were described by reporting their mean (SD). Median and inter-quartile ranges were reported for TGs levels because the distribution was highly positively skewed. All analyses were performed separately for males and females. The statistical significance of the differences in mean levels of normally distributed variables was examined using the t-test. The Kruskal-Wallis test was used for TGs. Chi-square test was performed to test univariate statistical association between categorical variables at baseline.

All baseline characteristics including age, educational level, marital status, smoking, systolic blood pressure, WC, diabetes, and history of CVD, were included in the logistic model with participation as the outcome. The probability of participation was estimated using logistic model and used as a propensity score. We added this propensity score to the longitudinal models as a covariate and examined if the probability of participation was associated with trend in lipid levels [24]. Furthermore we adjusted propensity score for all baseline characteristics in all four examination cycles and the parameter estimates remained essentially unchanged. Therefore, the selection bias is unlikely to have affected our estimations.

Trends of TC, LDL-C, HDL-C, and TGs levels were primary lipid measures of interest for the current analyses. We further explored the trend in the population levels of non-HDL-C, TC/HDL-C and TG/HDL-C ratio as well. Time trends in the population levels of the lipid measures, were examined using generalized estimating equations (GEE). The GEE method facilitates analysis of longitudinal data or repeated measures on dependent variables of many different distributions, mainly binary data [25]. GEE is a statistical technique enabling researchers to restrict modeling to the first moment which require only the correct working correlation matrix which specified the univariate marginal distributions [26]. Analysis with an exchangeable correlation structure with only random intercept was designed for the current data. Such an approach enabled us to account for the correlation among observations following assessment of each examination cycle. Models were developed at two hierarchial levels; age-adjusted (adjusting for age, propensity score and examination cycle) and multivariate-adjusted (adjusting for age, examination cycle, propensity score, BMI, current smoking, diabetes, hypertension and total cholesterol level [in analysis of HDL-C and TG]). Trends in BMI and FPG were also examined in both age and multivariate-adjusted models.

We also compared the trends in prevalence of high cholesterol, high TGs, low HDL-C, high non-HDL-C, high TC/HDL-C and high TG/HDL-C. The significance of trends in the proportions was examined by pooled logistic regression. All the analyses were repeated in the smaller sample (i.e. excluding individuals with prevalent or incident CVD and current use of lipid lowering drugs).

P-values < 0.05 were considered significant. All analyses were performed using SAS statistical software (ver. 9.2, SAS Institute, Car, NC, USA).

## Results

The comparison between baseline characteristics of participants with and without follow-up disclosed that the followed men had higher mean levels of TC, TGs and BMI, although they had lower rate of smoking. There was no difference in the lipid status between the followed versus non followed women; however, the followed participants had lower rate of smoking (Table 1).

**Table 1:** Baseline characteristics comparison of participants with and without follow-up with respect to different genders^*^

	**Male**			**Female**		
	**Follow up**	**No follow up**	**p-value**	**Follow up**	**No follow up**	**p-value**
	**(N = 3427)**	**(N = 968)**		**(N = 4687)**	**(N = 1284)**	
**Age (years)**	44.03 (15.4)	44.46 (17.37)	0.5	41.61 (13.85)	42.23 (16.4)	0.22
**Education**^**‡**^			0.6			0.43
**<6 yrs**	971 (28.4)	290 (30)		1909 (40.8)	540 (42.1)	
**6-12 yrs**	1870 (54.6)	513 (53.1)		2373 (50.7)	625 (48.8)	
**>12 yrs**	582 (17)	164 (17)		397 (8.5)	117 (9.1)	
**Marital status**^**‡**^			0.01			<0.001
**Single**	570 (16.6)	202 (20.9)		496 (10.6)	197 (15.3)	
**Married**	2829 (82.6)	758 (78.3)		3755 (80.1)	928 (72.3)	
**Divorce or widow**	28 (0.8)	8(0.8)		436 (9.3)	159 (12.4)	
**Current smoking**^**‡**^	923 (27.5)	319 (33.8)	<0.001	162 (3.5)	72 (5.7)	<0.001
**BMI (kg/m**^**2**^**)**	25.88 (4.07)	25.46 (4.25)	0.006	27.40 (5.38)	27.16 (5.83)	0.22
**Triglycerides (mmol/L)**^**†**^	156 (119)	139 (109)	<0.001	137 (112)	136 (110)	0.5
**HDL-C (mmol/L)**	38.42 (9.36)	38.36 (9.80)	0.87	45.06 (11.14)	45.02 (11.42)	0.92
**Cholesterol (mmol/L)**	205.45 (43.34)	200.73 (42.68)	0.004	212.95 (48.9)	212.52 (50.84)	0.78

*Abbreviations*: *BMI* body mass, *HDL-C* high-density lipoprotein cholesterol, *TG* triglycerides.

*Values are presented as mean (SD) for continuous parameters.

^†^Median (IQ).

^‡^n (%).

In the baseline examination, male participants were older, more likely to be single, being smokers, and had higher educational levels, as well as higher TGs, TC/HDL-C and TG/HDL-C levels compared to female participants; however mean BMI, total and HDL-C levels were higher in female participants in comparison with male ones (Table 2).

**Table 2:** Baseline characteristics participants by gender^*^

	**Male (N = 2085)**	**Female (N = 2866)**	**p-value**
**Age (years)**	43.8 (15.011)	41.61 (13.77)	<0.001
**Education**^**‡**^			<0.001
**<6 yrs**	566 (27.2)	1130 (39.5)	
**6-12 yrs**	1148 (55.1)	1479 (51.7)	
**>12 yrs**	368 (17.7)	251 (8.8)	
**Marital status**^**‡**^			<0.001
**Single**	362 (17.4)	283 (9.9)	
**Married**	1709 (82.0)	2330 (81.3)	
**Divorce or widow**	14 (0.7)	253 (8.8)	
**Current smoking**^**‡**^	568 (27.9)	118 (4.2)	<0.001
**BMI (kg/m**^**2**^**)**	25.73 (4.046)	27.51 (5.2)	<0.001
**Waist circumference (cm)**	88.53 (11.33)	87.62 (12.8)	0.011
**Systolic BP (mm Hg)**	120.71 (18.51)	118.45 (18.95)	<0.001
**Diastolic BP (mm Hg)**	78 (11.02)	77.82 (10.43)	0.6
**TC (mmol/L)**	5.3 (1.124)	5.51 (1.28)	<0.001
**HDL-C (mmol/L)**	1.0 (0.24)	1.166 (0.30)	<0.001
**TG (mmol/L)**^**†**^	1.75 (1.31)	1.54 (1.24)	<0.001
**Non-HDL-C (mmol/L)**	4.3 (1.13)	4.34 (1.28)	0.252
**TC/HDl-C**	5.6 (1.75)	5.0 (1.53)	<0.001
**TG/HDL-C**^**†**^	1.82 (1.06)	1.35 (1.34)	<0.001
**FPG (mmol/L)**	5.38 (1.5)	5.45 (1.97)	0.13

*Abbreviations*: *BMI* body mass, *BP* blood pressure, *FPG* Fasting plasma glucose, *HDL-C* high-density lipoprotein cholesterol, *non HDL-C* non high-density lipoprotein cholesterol, *TC* total cholesterol, *TG* triglycerides.

*Values are presented as mean (SD) for continuous parameters.

^†^Median (IQ).

^‡^n (%).

The baseline as well as follow-up characteristics of three assessments are shown in Table 3 in each gender. At baseline examination the mean age of participants were 43.8 (15.011) and 41.61 (13.77) years for men and women, respectively. Mean BMI was 25.73 (4.046) and 27.51(5.2) kg/m^2^ in men and women, respectively.

**Table 3:** Characteristics of participants by gender at examination cycle (baseline + 3 follow ups)^*^

	***Exam 1 (1999–2001)***	***Exam 2 (2002–2005)***	***Exam 3 (2005–2008)***	***Exam 4 (2008–2011)***
***Male***				
**Age (years)**	43.8 (15.011)	47.50 (14.15)	50.57 (14.14)	54.00 (14.16)
**BMI (kg/m**^**2**^**)**	25.73 (4.046)	26.57 (5.22)	27 (5.25)	27.23 (6.4)
**WC (cm)**	88.53 (11.33)	94.60 (10.65)	96.18 (10.23)	97.52 (10.76)
**Systolic BP (mm Hg)**	120.71 (18.51)	119.2 (17.55)	120.93 (18.91)	122.73 (18.26)
**Diastolic BP (mm Hg)**	78 (11.02)	75.58 (10.91)	76.57 (9.97)	79.91 (10.92)
**TC (mmol/L)**	5.3 (1.124)	5.02 (0.99)	4.97 (0.96)	4.96 (1.05)
**HDL-C (mmol/L)**	1.0 (0.24)	0.9 (0.21)	0.96 (0.21)	1.11 (0.24)
**TG (mmol/L)**^**†**^	1.75 (1.31)	2.05 (1.38)	2.01 (1.23)	1.9 (1.37)
**Non-HDL-C (mmol/L)**	4.3 (1.13)	4.11 (0.96)	4.00 (0.95)	3.83 (0.96)
**TC/HDL-C**	5.6 (1.75)	5.83 (1.63)	5.40 (1.5)	4.63 (1.35)
**TG/HDL-C**^**†**^	1.82 (1.06)	2.50 (1.86)	2.29 (1.7)	1.86 (1.46)
**FPG (mmol/L)**	5.38 (1.5)	5.56 (1.58)	5.51 (1.64)	5.86 (1.8)
**Current smoking**^**‡**^	568 (27.9)	489 (23.5)	553 (26.5)	555 (26.6)
**Lipid drug**^**‡**^	34 (1.66)	49 (3.2)	62 (3.77)	164 (9.63)
***Female***				
**Age (year)**	41.61 (13.77)	45.5 (13.21)	48.46 (13.13)	51.95 (13.15)
**BMI (kg/m**^**2**^**)**	27.51 (5.2)	29 (4.8)	29.2 (4.8)	30.23 (15)
**WC (cm)**	87.62 (12.8)	92.17 (12.28)	91.71 (12.36)	96.5 (12)
**Systolic BP (mm Hg)**	118.45 (18.95)	116.9 (19.42)	115.18 (19.92)	118.87 (21.08)
**Diastolic BP (mm Hg)**	77.82 (10.43)	75.51 (10.18)	73.05 (10.34)	76.68 (11.14)
**TC (mmol/L)**	5.51 (1.28)	5.23 (1.14)	5.17 (1.07)	5.2 (1.08)
**HDL-C (mmol/L)**	1.166 (0.30)	1.05 (0.26)	1.13 (0.27)	1.31 (0.28)
**TG (mmol/L)**^**†**^	1.54 (1.24)	1.85 (1.16)	1.81 (1.06)	1.73 (0.96)
**Non-HDL-C (mmol/L)**	4.34 (1.28)	4.17 (1.13)	4.04 (1.46)	3.88 (1.08)
**TC/HDL-C**	5.0 (1.53)	5.24 (1.7)	4.8 (1.46)	4.11 (1.19)
**TG/HDL-C**^**†**^	1.35 (1.34)	1.97 (1.63)	1.8 (1.38)	1.45 (1.07)
**FPG (mmol/L)**	5.45 (1.97)	5.57 (1.86)	5.55 (1.96)	5.9 (2.08)
**Current smoking**^**‡**^	118 (4.2)	159 (5.5)	141 (4.9)	160 (5.6)
**Lipid drug**^**‡**^	131 (4.64)	109 (4.83)	152 (6.5)	296 (12.38)

*Abbreviations:BMI* body mass index, *BP* blood pressure, *Exam* examination cycle, *FPG* Fasting plasma glucose, *TC* total cholesterol, *HDL-C* high-density lipoprotein cholesterol, *non HDL-C* non-high-density lipoprotein cholesterol, *TG* triglycerides, *WC* waist circumference.

*Values are presented as mean (SD) unless otherwise indicated.

†Median (IQ).

^‡^n (%).

As presented in the Tables 4 and 5, we observed a statistically significant decrease in the age and multivariate-adjusted levels of total and non-HDL cholesterol, TC/HDL-C and TG/HDL-C, as well as TGs for both genders (p-value for TGs trend was marginally significant for men). In contrast there was a significant increase in HDL-C levels across follow-up examinations in both men and women (all P-values <0.001). Meanwhile, the results demonstrated a significant increase in the population levels of BMI (in both genders) and FPG (only among men) in the same time frame in both genders.

**Table 4:** Age-adjusted mean levels^* ^**of fasting lipids, FPG and BMI by examination cycle in large sample**

	***Exam 1 (1999–2001)***	***Exam 2 (2002–2005)***	***Exam 3 (2005–2008)***	***Exam 4 (2008–2011)***	***p-value for trend***
***Male***					
**TC**^**†**^	5.22 (5.21-5.23)	5.12 (5.10-5.13)	4.99 (4.95-5.00)	4.88 (4.87-4.90)	<0.001
**HDL-C**^**†**^	0.943 (0.942-0.944)	0.982 (0.981-0.983)	1.016 (1.015-1.017)	1.052 (1.051-1.053)	<0.001
**TG**^**†**^	2.08 (2.07-2.09)	2.01 (2.00-2.02)	1.96 (1.95-1.97)	1.90 (1.89-1.91)	<0.001
**Non-HDL-C**^**†**^	4.28 (4.27-4.29)	4.13 (4.12-4.14)	3.97 (3.96-3.98)	3.82 (3.81-3.83)	<0.001
**TG/HDL-C**	2.45 (2.44-2.47)	2.28 (2.27-2.30)	2.15 (2.14-2.16)	2.00 (1.99-2.01)	<0.001
**TC /HDL-C**	5.81 (5.80-5.81)	5.48 (5.47-5.50)	5.17 (5.16-5.18)	4.85 (4.84-4.86)	<0.001
**FPG**^**†**^	5.35 (5.32-5.37)	5.54 (5.51-5.57)	5.62 (5.59-5.64)	5.73 (5.71-5.75)	<0.001
**BMI (kg/m**^**2**^**)**	25.88 (25.85-25.92)	26.36 (26.32-26.4)	26.87 (26.83-26.9)	27.37 (27.33-27.40)	<0.001
***Female***					
**TC**^**†**^	5.42 (5.40-5.44)	5.35 (5.33-5.37)	5.19 (5.17-5.21)	5.07 (5.05-5.09)	<0.001
**HDL-C**^**†**^	1.1004 (1.1002-1.1007)	1.1486 (1.1484-1.1487)	1.1960 (1.195-1.197)	1.2450 (1.244-1.246)	<0.001
**TG**^**†**^	1.86 (1.84-1.87)	1.84 (1.83-1.86)	1.76 (1.75-1.77)	1.71 (1.69-1.72)	<0.001
**Non-HDL-C**^**†**^	4.32 (4.30-4.34)	4.2 (4.18-4.22)	4 (3.98-4.02)	3.83 (3.81-3.85)	<0.001
**TG/HDL-C**	1.9 (1.88-1.91)	1.81 (1.80-1.82)	1.66 (1.64-1.70)	1.53 (1.52-1.54)	<0.001
**TC/HDL-C**	5.19 (5.17-5.21)	4.93 (4.91-4.95)	4.6 (4.57-4.61)	4.28 (4.26-4.30)	<0.001
**FPG**^**†**^	5.47 (5.44-5.49)	5.63 (5.60-5.66))	5.63 (5.61-5.66)	5.78 (5.75-5.81)	0.37
**BMI (kg/m**^**2**^**)**	27.73 (27.63-27.78)	26.64 (28.57-28.7)	29.24 (29.18-29.3)	29.94 (29.88-30.0)	<0.001

*Abbreviations:BMI* body mass index, *Exam* examination cycle, *FPG* fasting plasma glucose, *HDL-C* high-density lipoprotein cholesterol, *non HDL-C* non-high-density lipoprotein cholesterol, *TC* total cholesterol, *TG* triglycerides.

*Values are age adjusted means (95% confidence intervals) from generalized estimating equations to account for correlated observations. Adjusted for age, propensity score and examination cycle.

^†^mmol/L.

**Table 5:** Multivariate-adjusted mean levels^* ^**of fasting lipids, FPG and BMI by examination cycle in large sample**

	***Exam 1 (1999–2001)***	***Exam 2 (2002–2005)***	***Exam 3 (2005–2008)***	***Exam 4 (2008–2011)***	***p-value for trend***
***Male***					
**TC**^**†**^	5.21 (5.20-5.22)	5.13 (5.12-5.15)	5.00 (4.99-5.01)	4.88 (4.87-5.00)	<0.001
**LDL-C**^**†**^	3.82 (3.81-3.83)	3.73 (3.72-3.74)	3.58 (3.57-3.59)	3.43 (3.42-3.45)	<0.001
**HDL-C**^**†**^	0.95 (0.94-0.95)	0.97 (0.96-0.98)	1.013 (1.010-1.017)	1.058 (1.055-1.062)	<0.001
**TG**^**†**^	2.11 (2.08-2.14)	1.96 (1.92-1.99)	1.93 (1.90-1.96)	1.94 (1.91-1.98)	0.057
**Non-HDL-C**^**†**^	4.26 (4.25-4.28)	4.15 (4.14-4.17)	3.98 (3.97-4.0)	3.82 (3.80-3.83)	<0.001
**TG/HDL**	2.43 (2.41-2.46)	2.31 (2.3-2.34)	2.15 (2.12-2.17)	2.00 (1.97-2.03)	<0.001
**TC/HDL**	5.77 (5.75-5.8)	5.52 (5.5-5.54)	5.18 (5.16-5.2)	4.84 (4.81-4.86)	<0.001
**FPG**^**†**^	5.35 (5.32-5.37)	5.54 (5.51-5.57))	5.62(5.59-5.64)	5.73 (5.71-5.75)	<0.001
**BMI (kg/m**^**2**^**)**	25.92 (25.87-25.97)	26.3 (26.23-26.35)	26.78 (26.72-26.84)	27.45 (27.4-27.51)	<0.001
***Female***					
**TC**^**†**^	5.42 (5.39-5.43)	5.37 (5.34-5.4)	5.18 (5.16-5.20)	5.07 (5.04-5.06)	<0.001
**LDL-C**^**†**^	3.90 (3.88-3.92)	3.81 (3.79-3.83)	3.61 (3.59-3.63)	3.46 (3.44-3.48)	<0.001
**HDL-C**^**†**^	1.103 (1.101-1.104)	1.140 (1.130-1.142)	1.039 (1.037-1.194)	1.246 (1.241-1.251)	<0.001
**TG**^**†**^	1.88 (1.86-1.91)	1.80 (1.77-1.83)	1.71 (1.68-1.73)	1.74 (1.71-1.77)	<0.001
**Non-HDL-C**^**†**^	4.31 (4.29-4.33)	4.22 (4.20-4.24)	3.98 (3.96-4.00)	3.82 (3.80-3.85)	<0.001
**TG/HDL-C**	1.88 (1.87-1.90)	1.82 (1.80-1.84)	1.63 (1.61-1.65)	1.54 (1.51-1.57)	<0.001
**TC/HDL-C**	5.20 (5.17-5.21)	4.95 (4.93-4.98)	4.57 (4.56-4.60)	4.28 (4.25-4.32)	<0.001
**FPG**^**†**^	5.47 (5.44-5.49)	5.63 (5.60-5.66)	5.63 (5.61-5.66)	5.78 (5.75-5.81)	0.37
**BMI (kg/m**^**2**^**)**	27.76 (27.7-27.82)	28.64 (28.56-28.71)	29.11 (29.05-29.18)	30.02 (29.95-30.10)	<0.001

*Abbreviations:BMI* body mass index, *Exam* examination cycle, *FPG* fasting plasma glucose, *HDL-C* high-density lipoprotein cholesterol, *LDL-C* low-density lipoprotein cholesterol, *non HDL-C* non-high-density lipoprotein cholesterol, *TC* total cholesterol, *TG* triglycerides.

*Values are age adjusted means (95% confidence intervals) from generalized estimating equations to account for correlated observations. Adjusted for age, propensity score, examination cycle, BMI, current smoking, hypertension, Diabetes (except for FPG) and total cholesterol (in analyses of HDL-C and TGs), using covariates from the examination in question.

^†^mmol/L.

Table 6 show the time trends in the age and multivariate-adjusted prevalence rate of different aspects of dyslipidemia in the large sample. During the study, in both gender, there were significant decreases in the prevalence of hypercholesterolemia, hypertriglyceridemia, low HDL-C, high non-HDL-C, high TC/HDL-C and high TG/HDL-C. Furthermore consumption of lipid lowering drugs significantly (p < 0.001) increased in both genders.

**Table 6:** Proportions of participants in Lipid-related categories^* ^**by examination cycle in large sample**

	***Male***					***Female***				
	***Exam 1 (1999–2001)***	***Exam 2 (2002–2005)***	***Exam 3 (2005–2008)***	***Exam 4 (2008–2011)***	***p-value for trend***	***Exam 1 (1999–2001)***	***Exam 2 (2002–2005)***	***Exam 3 (2005–2008)***	***Exam 4 (2008–2011)***	***p-value for trend***
***Age adjusted***^***†***^										
**High cholesterol**	18.3	14.9	11.7	9.2	<0.001	24.8	22.1	17.3	14.2	<0.001
**Low HDL-C**	72.2	66.4	60.8	54.6	<0.001	46.2	39.4	32.9	27.1	<0.001
**High Triglycerides**	33.1	30.1	27.8	25.4	<0.001	26.0	25.2	22.6	21.0	<0.001
**High Non-HDL-C**	20.3	15.8	11.9	9.0	<0.001	23.1	19.2	13.8	10.3	<0.001
**High TG/HDL-C**	40.2	44.3	38.4	25.4	<0.001	28.3	25.9	22.3	19.6	<0.001
**High TC /HDL-C**	37.6	42.5	30.3	14.7	<0.001	26.8	21.1	14.9	10.7	<0.001
**Lipid drug (%)**	1.4	2.9	5.0	8.8	<0.001	3.6	5.8	7.8	11.2	<0.001
***Multivariate adjusted***^***‡***^										
**High cholesterol**	18.1	15.2	11.8	9.4	<0.001	24.5	22.5	17.2	14.1	<0.001
**Low HDL-C**	71.0	67.8	61.2	53.6	<0.001	45.4	40.6	33.1	26.5	<0.001
**High Triglycerides**	33.7	29.1	27.2	26.5	<0.001	26.6	24.4	21.3	21.9	<0.001
**High Non-HDL-C**	19.9	16.2	12.1	9.0	<0.001	22.9	19.7	13.6	10.2	<0.001
**High TG/HDL-C**	32.2	39.4	34.1	29.7	<0.001	28.0	26.8	21.8	19.5	<0.001
**High TC /HDL-C**	41.3	34.4	26.0	19.1	<0.001	26.7	21.8	14.8	10.6	<0.001
**Lipid drug (%)**	1.5	2.9	4.8	9.0	<0.001	3.7	6.1	7.6	11.4	<0.001

*Abbreviations:Exam* examination cycle, *HDL-C* high-density lipoprotein cholesterol, *non HDL-C* non-high-density lipoprotein cholesterol, *TC* total cholesterol, *TG* triglycerides.

Definitions: High cholesterol ≥6.19 mmol/L; Low HDL-C <1.036 mmol/L; High Triglycerides ≥2.26 mmol/L; High Non-HDL-C ≥5.15 mmol/L; High TG/HDL-C ≥2.18; High TC /HDL-C ≥5.97.

*Values are age and multivariate adjusted percentage from pooled logistic regressions including examination cycle to account for correlated observations.

^†^Adjusted for age, propensity score, and examination cycle.

^‡^Adjusted for age, propensity score, examination cycle, BMI, current smoking, hypertension, Diabetes (except for FPG) and total cholesterol (in analyses of HDL-C and TGs), using covariates from the examination in question.

As ancillary analyses we repeated all analyses among individuals without CVD who were not on lipid lowering drugs and observed that the results remained essentially unchanged in both age and multivariate-adjusted models (Tables 7, 8 and 9).

**Table 7:** Age-adjusted mean levels^* ^**of fasting lipids, FPG and BMI by examination cycle in small sample**

	***Exam 1 (1999–2001)***	***Exam 2 (2002–2005)***	***Exam 3 (2005–2008)***	***Exam 4 (2008–2011)***	***p-value for trend***
***Male***					
**TC**^**†**^	5.14 (5.13-5.16)	5.07 (5.06-5.09)	5.00 (4.98-5.01)	4.93 (4.91-4.94)	<0.001
**HDL-C**^**†**^	0.937 (0.935-0.938)	0.976 (0.975-0.978)	1.015 (1.014-1.017)	1.055 (1.053-1.056)	<0.001
**TG**^**†**^	1.97 (1.96-1.98)	1.94 (1.92-1.95)	1.91 (1.88-1.92)	1.87 (1.86-1.89)	<0.001
**Non-HDL-C**^**†**^	4.20 (4.19-4.22)	4.09 (4.08-4.10)	3.97 (3.96-3.99)	3.86 (3.85-3.87)	<0.001
**TG/HDL-C**	2.33 (2.31-2.34)	2.21 (2.19-2.22)	2.08 (2.07-2.10)	1.96 (1.95-1.98)	<0.001
**TC/HDL-C**	5.74 (5.73-5.76)	5.46 (5.44-5.47)	5.16 (5.15-5.18)	4.88 (4.86-4.9)	<0.001
**FPG**^**†**^	5.14 (5.12-5.16)	5.30 (5.28-5.32)	5.45 (5.44-5.47)	5.62 (5.60-5.63)	0.014
**BMI (kg/m**^**2**^**)**	25.85 (25.79-25.9)	26.36 (26.31-26.41)	26.85 (26.8-26.91)	27.37 (27.32-27.43)	<0.001
***Female***					
**TC**^**†**^	5.19 (5.16-5.21)	5.14 (5.12-5.17)	5.09 (5.07-5.11)	5.05(5.03-5.08)	<0.001
**HDL-C**^**†**^	1.0945 (1.094-1.095)	1.149 (1.148-1.150)	1.203 (1.202-1.204)	1.258 (1.257-1.259)	<0.001
**TG**^**†**^	1.63 (1.62-1.65)	1.62 (1.61-1.64)	1.61 (1.59-1.62)	1.60 (1.59-1.61)	<0.001
**Non-HDL-C**^**†**^	4.09 (4.07-4.11)	4.00 (3.97-4.02)	3.89 (3.88-3.91)	3.80 (3.77-3.82)	<0.001
**TG/HDL-C**	1.64 (1.63-1.66)	1.57 (1.56-1.58)	1.5 (1.48-1.5)	1.42 (1.40-1.43)	<0.001
**TC/HDL-C**	4.97 (4.95-5.00)	4.72 (4.70-4.74)	4.46 (4.44-4.48)	4.21 (4.19-4.23)	<0.001
**FPG**^**†**^	5.09 (5.07-5.11)	5.22 (5.20-5.24)	5.34 (5.32-5.36)	5.48 (5.46-5.50)	0.014
**BMI (kg/m**^**2**^**)**	27.43 (27.35-27.5)	28.16 (28.08-28.23)	28.81 (28.74-28.9)	29.50 (29.43-29.58)	<0.001

*Abbreviations:BMI* body mass index, *FPG* fasting plasma glucose, *exam* examination cycle, *TC* total cholesterol, *HDL-C* high-density lipoprotein cholesterol, *non HDL-C* non high-density lipoprotein cholesterol, *TG* triglycerides.

*Values are age adjusted means (95% confidence intervals) from generalized estimating equations to account for correlated observations. Adjusted for age, propensity score and examination cycle.

^†^mmol/L.

**Table 8:** Multivariate-adjusted mean levels^* ^**of fasting lipids, FPG and BMI by examination cycle in small sample**

	***Exam 1 (1999–2001)***	***Exam 2 (2002–2005)***	***Exam 3 (2005–2008)***	***Exam 4 (2008–2011)***	***p-value for trend***
***Male***					
**TC**^**†**^	5.13 (5.12-5.15)	5.08 (5.06-5.1)	5.00 (4.99-5.2)	4.93 (4.91-4.95)	<0.001
**HDL-C**^**†**^	0.940 (0.930-0.950)	0.970 (0.900-0.980)	1.012 (1.008-1.016)	1.058 (1.053-1.060)	<0.001
**TG**^**†**^	1.97 (1.95-1.99)	1.95 (1.93-1.98)	1.91 (1.88-1.94)	1.89 (1.86-1.91)	<0.001
**Non-HDL-C**^**†**^	4.19 (4.17-4.21)	4.11 (4.09-4.13)	3.99 (3.97-4.00)	3.86 (3.84-3.88)	<0.001
**TG/HDL-C**^**†**^	2.32 (2.29-2.35)	2.22 (2.19-2.26)	2.10 (2.07-2.13)	1.96 (1.93-1.99)	<0.001
**TC/HDL-C**	5.72 (5.69-5.75)	5.48 (5.45-5.51)	5.19 (5.16-5.22)	4.86 (4.83-4.90)	<0.001
**FPG **^**†**^	5.14 (5.12-5.16)	5.30 (5.28-5.32)	5.45 (5.44-5.47)	5.62 (5.60-5.63)	0.048
**BMI, (kg/m**^**2**^**)**	25.91 (25.84-25.99)	26.3 (26.22-26.37)	26.78 (26.7-26.86)	27.45 (27.35-27.53)	<0.001
***Female***					
**TC**^**†**^	5.18 (5.15-5.20)	5.15 (5.13-5.18)	5.09 (5.06-5.11)	5.05 (5.03-5.07)	<0.001
**HDL-C**^**†**^	1.094 (1.092-1.096)	1.144 (1.141-1.146)	1.202 (1.199-1.204)	1.256 (1.254-1.259)	<0.001
**TG**^**†**^	1.62 (1.60-1.64)	1.63 (1.61-1.65)	1.59 (1.57-1.61)	1.60 (1.58-1.62)	<0.001
**Non-HDL-C**^**†**^	4.08 (4.06-4.11)	4.01 (3.98-4.03)	3.88 (3.86-3.91)	3.80 (3.77-3.82)	<0.001
**TG/HDL-C**^**†**^	1.63 (1.61-1.65)	1.6 (1.57-1.60)	1.47 (1.45-1.49)	1.42 (1.40-1.44)	<0.001
**TC/HDL-C**	4.96 (4.94-5.00)	4.75 (4.72-4.77)	4.46 (4.43-4.48)	4.21 (4.19-4.23)	<0.001
**FPG **^**†**^	5.10 (5.08-5.12)	5.24 (5.21-5.26)	5.34 (5.31-5.36)	5.50 (5.48-5.50)	0.05
**BMI, (kg/m**^**2**^**)**	27.46 (27.38-27.54)	28.15 (28.07-28.23)	28.73 (28.65-28.81)	29.58 (29.5-29.67)	<0.001

*Abbreviations:BMI* body mass index, *exam* examination cycle, *FPG* fasting plasma glucose, *TC* total cholesterol, *LDL-C* low-density lipoprotein cholesterol, *HDL-C* high-density lipoprotein cholesterol, *non HDL-C* non high-density lipoprotein cholesterol, *TG* triglycerides.

*Values are age adjusted means (95% confidence intervals) from generalized estimating equations to account for correlated observations. Adjusted for age, propensity score, examination cycle, BMI, current smoking, hypertension, Diabetes (except for FPG) and total cholesterol (in analyses of HDL-C and TGs), using covariates from the examination in question.

^†^mmol/L.

**Table 9:** proportions of participants in Lipid-related categories^* ^**by examination cycle in small sample**

	***Male***					***Female***				
	***Exam 1 (1999–2001)***	***Exam 2 (2002–2005)***	***Exam 3 (2005–2008)***	***Exam 4 (2008–2011)***	***p-value for trend***	***Exam 1 (1999–2001)***	***Exam 2 (2002–2005)***	***Exam 3 (2005–2008)***	***Exam 4 (2008–2011)***	***p-value for trend***
***Age adjusted***^***†***^										
**High cholesterol**	15.6	13.2	11.2	9.4	<0.001	18.0	16.4	14.6	13.4	<0.001
**Low HDL-C**	72.4	66.7	60.5	53.8	<0.001	46.8	39.2	32.1	25.7	<0.001
**High Triglycerides**	31.2	27.9	27.3	24.6	<0.001	18.3	17.1	18.1	16.8	<0.001
**High Non-HDL-C**	17.3	13.9	11.1	8.8	<0.001	16.1	13.1	10.4	8.4	<0.001
**High TG/HDL-C**	42.6	38.0	33.7	29.4	<0.001	21.4	19.5	17.5	15.9	<0.001
**High TC/HDL-C**	40.4	32.2	24.9	18.8	<0.001	21.4	16.1	11.7	8.6	<0.001
***Multivariate adjusted***^***‡***^										
**High cholesterol**	15.5	13.4	11.4	9.7	<0.001	17.7	16.6	14.7	13.5	<0.001
**Low HDL-C**	71.7	67.0	60.9	53.0	<0.001	42.2	40.0	32.1	25.4	<0.001
**High Triglycerides**	31.1	28.0	27.6	24.7	<0.001	18.1	17.1	17.9	16.6	<0.001
**High Non-HDL-C**	17.0	14.2	11.3	9.0	<0.001	15.9	13.4	10.4	8.5	<0.001
**High TG/HDL-C**	42.1	38.7	34.8	29.4	<0.001	21.2	19.9	17.2	15.9	<0.001
**High TC/HDL-C**	39.8	33.0	25.4	18.6	<0.001	21.2	16.5	11.7	8.4	<0.001

*Abbreviations:exam* examination, *TC* total cholesterol, *HDL-C* high-density lipoprotein cholesterol, *non HDL-C* non high-density lipoprotein cholesterol, *TG* triglycerides.

Definitions: High cholesterol ≥6.19 mmol/L; Low HDL-C <1.036 mmol/L; High Triglycerides ≥2.26 mmol/L; High Non-HDL-C ≥5.15 mmol/L; High TG/HDL-C ≥2.18; High TC /HDL-C ≥5.97.

*Values are age and multivariate adjusted percentage from pooled logistic regressions including examination cycle to account for correlated observations.

^†^Adjusted for age, propensity score, and examination cycle.

^‡^Adjusted for age, propensity score, examination cycle, BMI, current smoking, hypertension, Diabetes (except for FPG) and total cholesterol (in analyses of HDL-C and TGs), using covariates from the examination in question^‡^Adjusted for age, propensity score, examination cycle, BMI, current smoking, hypertension, Diabetes (except for FPG) and total cholesterol (in analyses of HDL-C and TGs), using covariates from the examination in question.

## Discussion

Using a large cohort of a Middle Eastern adults population, we demonstrated favorable time trends in the population levels of total cholesterol, HDL-C, non-HDL-C, TG, TG/HDL-C and TC/HDL-C among both genders during 10 years follow up. Such favorable trends in lipids levels and ratios could not be fully accounted for by the significant increase in consumption of lipid lowering drugs. In contrast, however, population levels of general adiposity and FPG have increased in both genders potentially leading to long term risk of cardiovascular disease.

Cardiovascular disease has long been known to be a multi-factorial disease. In 1948, the Framingham Heart Study embarked on an ambitious project in health research to identify the common factors that contribute to cardiovascular disease [27], over the years, careful monitoring of the Framingham Study population has led to the identification of the major CVD risk factors among which lipid measures have been widely investigated [28,29]. Several initiatives have been launched in different countries to reduce the burden of CVD by reducing the level of its risk factors [30-32]. As such, the important question to be answered would be whether these findings could be translated to better risk factor levels in the population [33]. Perceiving this need, several studies have attempted to explore the time trends in the CVD risk factors in the populations of different ethnic groups. Favorable trends in the lipid measures have been documented by large studies conducted in European as well as North American populations [28,34,35]. However, the effects of these favorable trends on the population burden of CVD have paralleled the increasing trends in the obesity and diabetes [36,37]. As a consequence, the favorable trends in the CVD morbidity and mortality leveled off at beginning of the 21st century [11,35]. Middle Eastern population has been estimated to harbor a great fraction of the world’s burden of diabetes and obesity [20,38]. In fact, while America have been estimated to spend more than half of the global health expenditure on diabetes, less than 10% of the global health expenditure will be spent in the low and middle-income countries [39]. Therefore, resorting to controlling other risk factors that are more amenable to treatment and prevention continues to be the best policy to stopping CVD. We demonstrated statistically significant and clinically meaningful favorable trends in the population levels of the lipid measures over the last decade, finding that did not change after multivariate adjustment and elimination of lipid lowering drugs users. The desirable trends for lipid measures in our population is compatible with those of other studies using cross-sectional surveys [9-11,14] and prospective studies [13,16-18] as well.

We observed an increase in the population levels of HDL-C simultaneously with decline in triglycerides levels, a finding in agreement with results of the Framingham study [13] and in contrast to those of other studies [9,10,12], showing simultaneous increases in TC, TGs and decreasing HDL-C.

In our study the prevalence of high cholesterol decline about 48% and 42% in men and women respectively, while the percentage of US adults with high total cholesterol decline by 27% between 1999 and 2010; furthermore it was reported that about 12% of female participants and 31% of male participants had low HDL-C [14]. However, in our adult population the prevalence of low HDL-C was 52% for men and 26% for women, despite decreasing trend in low HDL-C, dyslipidemia still has a higher prevalence, compared to U.S adults.

Although changes in nutritional habits [40,41], physical activity and endurance exercise [42,43] are all known to be among important determinants of serum lipid levels; the decreasing trends in lipid levels in our population could hardly be explained by life style changes (i.e. physical activity), since it was shown that low physical activity is common in Iranian population [44,45]. It has been shown, however, that over 30% of Iranian families are now consuming less hydrogenated oil than they did in the past [46,47], that could possibly explain the favorable lipid trend in TLGS population during recent years. In line with our findings, cross-sectional National studies conducted by Ministry of Health and Medical Education among Iranian adult population in whole country, showed significant decrease in level of high total cholesterol (Etemad K., Center for Non-communicable Diseases Control, Ministry of Health and Medical Education, Tehran, Iran, unpublished observations).

Another factor that affected serum lipids is cigarette smoking. Craig et al. in a meta-analysis about effect of smoking on cardiovascular risk factors demonstrated that compared with non-smokers, cigarettes smokers had significantly higher TC, TG and lower concentrations of HDL-C [48]. The review study in field of smoking between 1991 and 2007 in Iran showed that during these years smoking did not increased, which might justify the favorable trend in HDL-C level of our population [49].

Our study has both strengths and limitations. The strengths of the current study lie in its design as a long term community-based prospective study conducted on a large sample of Middle Eastern men and women, a region where data on secular trends in the lipid levels is lacking, also lipid profile components were measured rather than self-reported. Our findings need to be interpreted in light of its limitations as is inherent to any prospective study [13]. First, survivor bias might have biased favorable trends towards overestimated values, i.e. individuals with possible unfavorable changes in their lipid levels might have died and thus been excluded from repeated measurements. Second, as any cohort study we cannot rule out healthy cohort effect i.e. the possibility of the effect of knowledge about the serum lipids might have affected the lifestyle or lipid drugs consumption in the participants, leading to the favorable trends in lipid levels. Third, we did not have any systematic data on the trends of nutritional behavior, physical activity and knowledge of the primary prevention in our population, consequently it is not possible to test the hypothesis that whether the trends observed could be attributable to changes in physical activity or nutrition status. Forth, the results obtained in the current study might not applicable to certain age groups including younger (less than 20 years) and older (over than 75 years) ones. Finally, our population was selected from middle-aged Middle East Caucasians and therefore we cannot make inferences beyond a similar group.

## Conclusion

The main findings in this study include an observed decrease in total cholesterol, LDL-C, triglyceride levels as well as TC/HDL-C and TG/HDL-C in an adult Iranian population during the period of 1999–2001 until 2008–2011.Overall the trends of general obesity and FPG level were observed to have increased. The net effect of such trends on the CVD burden warrants further investigations.

## Competing interests

The authors declare that they have no competing interests

## Authors’ contributions

Conceived and designed the experiments: FH, MK. Analyzed the data: SA. Wrote the paper: MK, FH, ML, MB, involved in acquisition of analysis data files from cohort database: SA, Contributed to interpretation of results: FH, MK, NS, MT, FA, ML, MB. Critically reviewed drafts of the manuscript and made comments to improve clarity: MK, FH, ML, and MT. All authors read and approved the final manuscript.
